# Supplementary material for: Are carbon emissions trading and green financial instruments synergistic? -Comprehensive quantitative research based on content analysis
Source: PLoS One. 2024 Mar 7;19(3):e0298601. doi: 10.1371/journal.pone.0298601 (PMC10919601; doi:10.1371/journal.pone.0298601)
Supplement: S1 Data — (ZIP) [file pone.0298601.s001.zip › Data/地级市Z_F工作报告.zip!/╔╜╢1⁄2/║╩╘≤╩╨/└┤╫╘╣1⁄2╓┌║┼í╛┬φ┐╦╩2╛▌═°í┐_2016.docx]

各位代表：

　　现在，我代表市人民政府向大会报告工作，请予审议，并请市政协各位委员提出意见。

　　一、“十二五”经济社会发展规划实施情况

　　2011年至2015年，全市人民开拓创新、扎实苦干，奋力推进经济建设、政治建设、文化建设、社会建设、生态文明建设，圆满完成了“十二五”规划确定的任务目标，在科学赶超的征程中迈出了坚实步伐，全市经济社会发展站在了新的历史起点上。

　　——过去的五年，是我市经济加快发展、综合实力大幅提升的五年。地区生产总值由1227亿元增加到2400亿元，公共财政预算收入由84.7亿元增加到177.7亿元，金融机构年末存款余额由1092.6亿元增加到2550.5亿元，规模以上固定资产投资由583亿元增加到1074亿元，社会消费品零售总额由666亿元增加到1350亿元，进出口总额由18.2亿美元增加到44.2亿美元。

　　——过去的五年，是我市转变发展方式、质量和效益显著提高的五年。三次产业比由17.8:52.9:29.3调整为11.3:52.9:35.8。新增市级以上高新技术企业、创新型企业244家，省级企业技术中心、工程实验室、工程技术研究中心50家，中国驰名商标9个、山东省著名商标和名牌产品65个。高新技术产业产值占比由25.9%提高到31.6%。粮食产量连创历史新高，农村土地流转面积占比由2.6%提高到17.7%，农田有效灌溉面积占比由63.4%提高到81.6%，耕种收综合机械化水平由78.6%提高到86%，农业科技进步贡献率由49%提高到56%。一批支撑性现代服务业项目相继建成投用。旅游业健康发展，新增4A级旅游景区4个，被评为中国优秀旅游城市。

　　——过去的五年，是我市城市功能不断增强、城乡面貌快速改变的五年。常住人口城镇化率由35%提高到44.6%。市区新建改造道路72条、136公里，新增供热面积500万平方米，配套建设雨污管网240公里，环卫保洁面积新增197万平方米，绿化覆盖率达到40.9%，被评为国家园林城市。8个县城建成区面积扩大44.6平方公里。建成农村新型社区372个，新建改建农村公路7006公里、农村供电线路9864公里，新增农村饮水安全人口393.2万人，城乡环卫一体化实现全覆盖。

　　——过去的五年，是我市人民群众收入稳步增长、生活明显改善的五年。城乡居民人均可支配收入由11584元、4970元增加到20330元和9820元。146万农村贫困人口实现脱贫。企业退休人员月人均养老金由1276元提高到2182元，城市低保人均月保障标准、农村低保年保障标准分别由240元、1400元提高到380元和2800元，五保集中供养和大病救助封顶线分别由2100元、7000元提高到4200元和22000元。城乡居民人均住房面积分别由34.4平方米、31.9平方米提高到38.4平方米、39.7平方米。城乡居民每百户拥有汽车分别由9辆、1辆提高到27辆、10辆。人均预期寿命由77岁提高到79岁。

　　——过去的五年，是我市社会事业长足进步、经济社会协调发展的五年。新增城镇就业47万人，农村劳动力转移就业71万人。新建中小学校舍327.7万平方米，新建、改扩建城区义务教育学校129所，新建幼儿园1016所，新招录教师11037人。新建、改扩建公立医院26家，床位由25697张增加到36317张。每千名老年人拥有社会化养老床位由13张增加到31张。村级健身项目覆盖率达到63%。

　　——过去的五年，是我市社会治理持续加强、和谐创建卓有成效的五年。积极推进政府治理现代化。社会信用体系建设纵深推进。行政复议体制改革任务基本完成。社区网格化管理全面加强，平安创建活动深入开展，安全生产事故起数、死亡人数分别下降80.5%和60.3%。精神文明创建持续推进，社会主义核心价值观和“崇文尚德、务实图强”的菏泽精神得到弘扬。军民“双拥共建”深入开展，被评为“全国双拥模范城”。

　　二、2015年主要工作任务完成情况

　　刚刚过去的一年，我们以党的十八大和十八届三中、四中、五中全会精神为指针，认真贯彻落实习近平总书记系列重要讲话特别是视察山东及菏泽重要讲话精神，在中共菏泽市委的正确领导下，主动适应经济发展新常态，深入实施产业提升、基础突破、扶贫攻坚、和谐创建“四项工程”，突出培植五大主导产业，着力构建新机制、激发新活力、培育新动能，经济社会持续健康较快发展，圆满完成了市十八届人大四次会议确定的目标任务。预计全年完成地区生产总值2400亿元，增长9.3%；公共财政预算收入实现177.7亿元，增长9.7%；城乡居民人均可支配收入达到20330元、9820元，分别增长8.3%、10%；金融机构年末存贷款余额达到2550.5亿元、1635.4亿元，分别比年初增加322.2亿元、188.3亿元。

　　（一）着力培育增长新动能，发展亮点不断涌现。积极适应经济发展新常态，大力培植新的增长点，迅速构筑竞争新优势，提升了经济活跃度，膨胀了总量，增强了后劲。强力推进精准招商和项目建设。全年引进落地项目410个，增长12.3%。储备项目1273个，11个项目列入省重点。200个市重点项目完成投资550亿元，规模以上固定资产投资完成1074亿元。扎实推动全民创业。采取出台政策、强化培训、搭建平台、优化服务、设立“市长创业奖”等措施，形成推动竞相创业的整体合力。全市新登记注册各类市场主体14.9万户、增长78.3%，其中新增个体户12.1万户、私营企业2.4万户，分别增长88.4%、147.2%，创业带动就业9万多人。“四上”企业达到5971家，新增889家。积极发展外向型经济。被评为“国家级出口林产品质量安全示范区”、“山东省牡丹生产加工出口优质产品生产基地”。海关场站建设实现新突破。东明石化成为全国第一家获得进口原油使用资质、原油非国营贸易进口资质和成品油出口资质的地炼企业。全市完成进出口44.2亿美元，增长25.6%。实际利用外资2.3亿美元，增长5%。大力发展电子商务。与阿里巴巴集团签署了全面战略合作协议，以农特产品为主的“特色中国菏泽馆”、以工业品为主的“1688菏泽产业带”等一批电商平台上线运营，“千县万村”试点县区发展到5个。培训电商人员8.3万人次。全省电子商务示范县、“淘宝镇”、“淘宝村”发展到2个、4个、35个，分别占全省的1/5、2/3、1/2以上。发展电商园区16个，电商企业达到2.5万家，从业人员22万人，电商交易额达到700亿元、增长40%以上。

　　（二）加快转变发展方式，发展质量明显提高。以质量和效益为中心，大力实施“产业提升”工程，推动了转型发展。着力推进工业转型。着眼规模膨胀、链条延伸、转型升级，积极推动四大主导产业集群集聚发展，主营业务收入达到6340亿元，增长9.2%。大力实施百强企业培育计划，规模效益大幅提升，纳税前100家工业企业入库税金84.2亿元，同比增长24%。大力实施品牌战略，新增中国驰名商标2个、山东省著名商标11个、山东名牌产品10个。积极推进现代农业建设。以“一村一品”为抓手，大力推进农业内部结构和种植业结构调整。粮食生产实现“十二连增”。新增土地流转面积43万亩、规模以上农副产品加工企业174家、农民专业合作社2825家、家庭农场1129家、省级农业标准化生产基地10万亩、省级以上畜禽标准化示范养殖场38家、省级以上“一村一品”示范村镇12个。切实做大做强服务业。大力发展商贸物流业、现代服务业，新兴服务业占比首次超过传统服务业。纳税前100家非工业企业入库税金42.2亿元，同比增长22.1%。加快创新驱动发展。与清华大学、山东大学、山东工艺美院、山东省科学院等展开了全面战略合作，举办了“高层次人才菏泽行”活动。220多家企业与高等院校、科研单位建立了稳定的合作关系。在建、建成科技孵化器12个，在孵项目162个。新增院士工作站4家、市级以上高新技术企业57家。全社会研发投入占地区生产总值的比重达到1.22%，高新技术产业产值占比达到31.6%。坚持绿色低碳发展。淘汰落后设备1091台套，节能30万吨标煤。盘活利用低效工业用地7672亩，治理采煤塌陷区1.3万亩。国家级、省级生态乡镇分别达到44个、109个。城市环境空气质量同比改善6%；蓝天白云天数186天，同比增加41天。三条主要河流出境断面水质稳定达标。全面完成省下达的各项约束性指标。

　　（三）大力实施基础突破，城镇承载能力得到增强。实施了8大类29项重大基础设施建设。菏泽机场配套工程建设已经启动，鲁南快速铁路客运通道曲阜经菏泽至兰考段可研报告正在完善，菏泽至徐州铁路完成方案研究，德商高速鄄菏段建成通车，洙水河航道实现全线试通航。齐鲁工业大学菏泽校区、华润东明热电联产、新万福河航道等项目开工建设。10座平原水库主体工程基本完工，配套建成9座净水厂、铺设供水骨干管网1140公里。扎实推进市区重点工程建设，一大批项目建成投用。埋设雨污管网30公里、供热管网29.3公里、天然气管道35公里。县城和重点镇建设步伐加快，“四点对接”的新型城镇体系逐步形成。

　　（四）积极推进各项改革，为发展注入了活力和动力。加快政府职能转变，编制完成市级行政权力清单、责任清单，压缩审批时限70%以上。实施商事制度改革，营商环境持续优化。改革了市区城市规划建设管理、卫生计生、住房建设、食品药品、工商质监等体制。事业单位分类改革扎实推进。完成规范化公司制改造企业23家。农村产权制度改革取得新进展。稳步推进财税金融体制改革，“营改增”、公共资源市场化配置、农信社银行化等改革取得新成效。深化投融资体制改革，设立了鲁信基金、金源浦发科技发展基金、城市建设发展基金、牡丹产业基金，牡丹保险公司创建已进入审批程序。积极推进政府和社会资本合作，201个项目列入省项目库，总投资1587.7亿元。创新实施重点经济工作考核办法，发挥了积极的激励和导向作用。

　　（五）扎实办好民生实事，群众满意度进一步提升。民生支出占公共财政预算收入的比重达到77.7%。年初确定的10件民生实事全部完成。投资8.3亿元，解决了169.7万农村居民和18.7万农村师生的安全饮水问题。市老年大学、老干部活动中心和全民健身中心按期开工。新投入公共自行车1000辆、新能源公交车300辆。市创业实训孵化基地建成投用。为53万名高血压患者、17.3万名糖尿病患者免费建立了健康档案，开展了保健医疗服务。开工保障性安居工程23976套，改造农村危房7276户。建设农村公路1148.5公里。24.3万亩高标准农田实现刷卡取水灌溉。社会保障不断完善。实现减贫43万人。新增城镇就业9.9万人、农村劳动力转移就业20.1万人，城镇登记失业率3.12%。企业基本养老金人均月增加272元，居民基础养老金人均月提高到85元。市级机关和县区的工资都有较大幅度提高。社会事业全面发展。完成“全面改薄”投资31.4亿元，实施校舍建设类项目160.6万平方米，新建、改扩建城区学校25所，新建幼儿园224所。新增医疗建筑面积25万平方米、床位3000张。新建村级综合性文化服务中心700个、文化广场1605个。社会治理成效显著。刑事案件发案量下降6.6%。安全生产事故起数、死亡人数同比下降4.7%和13.1%。

　　（六）全面加强自身建设，政府机关作风不断改进。制定了《关于进一步加强自身建设的意见》，深入开展“三严三实”专题教育，努力形成干事创业的浓厚氛围。将233项目标任务逐一分解到政府领导班子成员及相关部门，向社会公开，明确责任，强化监督，促进了工作落实。完善重大行政决策程序，依法行政和决策水平进一步提高。自觉接受人大监督，加强与政协的民主协商，认真办理人大代表议案和政协委员提案。建立了市政府与人大代表联系制度。积极推进政务公开。严格落实党风廉政建设责任制，廉政建设进一步加强。

　　妇女儿童、关心下一代、残疾人、老龄、慈善等事业加快发展，民族宗教、外事侨务、人民武装、防震减灾、邮政通讯、气象、人防、统计、物价、对台、史志、档案等工作取得新的成绩。

　　各位代表，我市取得的每项成绩，都是认真贯彻党的十八大和十八届三中、四中、五中全会精神的结果，是省委、省政府和中共菏泽市委正确领导的结果，是市人大、市政协和社会各界大力支持的结果，是全市人民奋力拼搏的结果。在此，我谨代表市人民政府，向勤劳智慧的全市人民，向各级人大代表、政协委员，向各民主党派、工商联、人民团体和社会各界人士，向离退休老领导、老同志，向驻菏部队、武警官兵、公安干警，向所有关心支持菏泽发展的同志们、朋友们，表示衷心的感谢并致以崇高的敬意！

　　各位代表，我们清醒地认识到，我市经济社会发展中还存在一些困难和问题：经济结构不合理不协调的问题比较突出，企业整体创新能力不强，大多产品处于产业价值链的中低端；生态环境特别是大气污染问题仍然突出，在强化治理上还缺乏壮士断臂的勇气和措施；基本公共服务还不够均衡，城乡差别、城镇内部“二元结构”还比较明显；城市管理水平与城市发展及人民群众的要求还不够适应；脱贫攻坚的任务十分艰巨；政府系统工作人员的工作作风还有待进一步转变。对这些问题，我们一定高度重视，并努力加以解决。

　　三、“十三五”时期经济社会发展的目标任务

　　“十二五”是菏泽人民激情跨越、倾情书写华章的五年。从今年开始，我们步入了“十三五”发展的新阶段。这一时期，是全面建成小康社会的决胜阶段，也是我市实现“科学赶超、后来居上”奋斗目标的关键时期。谋划“十三五”，我们科学制定了奋斗目标：地区生产总值年均增长8.5%左右，公共财政预算收入年均增长10%左右，规模以上固定资产投资年均增长13%左右，社会消费品零售总额年均增长11%左右，城镇和农村居民人均可支配收入年均分别增长8.5%和9%左右，进出口总额年均增长12%左右。

　　决战“十三五”，我们面临千载难逢的机遇。一是经济发展长期向好。全国经济稳中向好的大趋势没有改变，支撑菏泽经济持续增长的基础和条件更加巩固，后发优势凸显。二是政策效应叠加。中央和省相继推出了一系列稳增长的政策，与中原经济区、西部隆起带等政策相互叠加，在政策、资金、项目、人才等方面给予我市大力倾斜。三是重大基础设施密集实施。机场、高铁、铁路、高速公路、航道、电力等一大批基础设施将在“十三五”期间建成，城市的综合承载能力将显著提升。四是扶贫攻坚带动。中央和省委省政府向全国、全省人民发出了扶贫攻坚决战决胜的动员令，出台了一系列政策措施，我们将集中受益。五是主导产业发展势头良好。生物医药、能源化工、机电设备制造、农副产品加工和商贸物流五大主导产业规模快速膨胀，为科学赶超提供了产业支撑。六是发展动力愈加强劲。习近平总书记亲临菏泽视察，对我们提出了后来居上的殷切希望，极大振奋了全市人民不甘落后、奋勇赶超的信心，激发了各方面的积极性和创造性，形成了干事创业、加快发展的共识共为。我们必须勇于抢抓机遇、善于把握机遇，发扬时不我待、只争朝夕的精神，凝心聚力，扎实苦干，在全面建成小康社会的征程中砥砺前行。

　　四、2016年的目标任务和工作重点

　　今年是“十三五”开局之年。根据中央和省市委的决策部署，牢固树立“创新、协调、绿色、开放、共享”发展理念，全面落实宏观政策要稳、产业政策要准、微观政策要活、改革政策要实、社会政策要托底的总体思路，实施去产能、去库存、去杠杆、降成本、补短板五大任务，加快结构性改革和体制机制创新，着力提高发展的质量和效益。

　　主要预期目标是：地区生产总值增长8.5%左右，公共财政预算收入增长9%左右，规模以上固定资产投资增长13%左右，规模以上工业增加值增长11%左右，社会消费品零售总额增长11%左右，进出口总额增长15%左右，利用外资增长5%左右，城镇和农村居民人均可支配收入增长9%左右、10%左右，城镇登记失业率控制在4%以内，全面完成省下达的节能减排约束性指标。

　　（一）牢牢扭住加快发展不放松，着力培育增长新动能，千方百计膨胀经济总量。我市经济总量小，存量调整的空间有限，优化经济结构的关键和出路在于扩大优质增量，集中精力把“蛋糕”做大。一是强力推进精准招商和项目建设。按照加强供给侧结构性改革的要求，以“投资有效益、产品有市场、企业有利润、员工有收入、政府有税收”为目标，专心专业策划项目，倾情倾力组织招商。把策划储备项目作为精准招商的前提，立足区位、资源、产业优势，加强投资机会研究，着力策划一批优质项目，每个县区策划储备过10亿元项目5个以上，全市纳入省重点的项目数量争取居全省前列。突出抓好资源招商、以商招商、产业链招商、盘活存量招商，切实提高招商的精准性和成功率，力争当年签约项目落地率达到50%以上。加快推进项目落地建设，促进早开工、早投产、早达效。力争完成规模以上固定资产投资1200亿元以上。二是培育壮大现有企业。坚持分类指导、精准施策，促进现有企业做大做强。继续抓好百强企业培育，推动大型企业集团、高成长性企业和优势企业膨胀提升，发挥引领和示范作用。尊重、关心、保护企业家，搞好企业负责人专项培训，提升企业管理团队的综合素质；大力支持企业家干事创业，促进企业不断扩大规模、提高核心竞争力，确保规模以上企业主营业务收入增长10%以上。积极稳妥处置丧失自我修复能力和自我发展能力的僵尸企业，提高资源配置效率，优化市场秩序。三是大力推动全民创业。认真落实促进全民创业的优惠政策，大力营造“人人想创业、个个能创业”的社会氛围。搭建众创、众包、众扶、众筹等支撑平台，强化创业培训实训、创业实践孵化，鼓励支持城乡居民就地就近创业，高校毕业生、退役士兵自主创业。突出抓好在外成功人士、农民工返乡创业，在市外建立返乡创业服务站，在市内依托存量资源建设返乡创业园，精心“筑巢引凤”，大力发展“归雁经济”，推动更多创业创新主体不断涌现。力争年内建成专业创业园区、综合性众创空间20家以上，新增各类市场主体7.5万户，其中新注册企业1.2万家。四是努力扩大有效投资。本着优化投向、加快投速、强化投效的原则，抓好政府与社会资本合作项目的策划推介，力争年内新落地50个，新进入省项目库80个。用足用活财税体制改革政策，大力压减“三公经费”支出，腾出更多资金设立政府引导基金，采取财政贴息、财政奖补、过桥贷款、风险补偿等办法，组织开展好“一对一”的银企对接活动。引导金融机构创新金融产品，扩大信贷投放，鼓励企业拓展直接融资渠道，确保新增社会信用总量500亿元以上。支持优势企业发行债券、公开募股，争取5家企业完成上市或挂牌。五是积极培育“四上”企业。实施企业梯次培植计划，出台激励政策，促进“个转企、小升规、规改股、股上市”，促使更多项目及时进入“四上”行列，力争新增“四上”企业600家以上。

　　（二）突出创新驱动，加快转型升级，不断提高经济发展的质量和效益。把创新作为引领发展的第一动力，突出抓好互联网与实体经济的深度融合，促进产业转型升级、提档增效。一是加大人才引进培养促转型。认真落实省委组织部等九部门出台的支持菏泽市人才发展的若干政策，加快破解我市人才特别是高层次人才缺乏的瓶颈制约。加快搭建科研工作站、工程技术研究中心等人才施展才华的空间。设立用好人才专项资金，组织实施好“金蓝领”培训、业务骨干访学研修计划，加快推进高技能人才培训基地、技师工作站建设。确保引进培养硕士学位以上和专业技术人才2300人，新增高技能人才4000人以上。二是做优做强工业促转型。以主导产业为重点，以高端高质高效发展为方向，积极融入中国制造2025，大力实施工业强基工程和品牌兴市战略。认真落实主导产业技术改造导向计划，确保完成技改投资360亿元、增长12%。以“1688菏泽产业带”为抓手，大力实施“千企触网”行动，引导企业积极应用物联网、云计算、大数据等信息技术，创新生产方式、运营模式。抓好公共研发平台建设，深化产学研合作，提高原始创新、协同集成创新、消化吸收再创新能力。力争年内建成省级以上标准科技孵化器4家，新增高新技术企业50家，高新技术产业产值占比提高1个百分点以上。引导和促进企业加大研发投入，力争规模以上企业研发投入强度达到1%以上，全社会研发投入占GDP的比重达到1.3%以上。引导各县区把各自的省级开发区作为转型发展的重要载体，大力实施公共设施优化提升工程，配套建设电商产业园、返乡创业园、中小企业孵化园、农业科技园。通过“一区四园”建设，推进三产融合、“三化”互动，加快培育壮大特色产业集群，打造转型升级发展的新优势。三是着力发展高效农业促转型。树立大农业、大食物理念，研究制定发展精致高效农业的意见，推动粮经饲统筹、农林牧渔结合、种养加一体、一二三产融合发展，提升农业的产出水平和比较效益。建立农村土地流转市场，完善流转机制，推动工商资本参与现代农业发展，促进土地向种植大户、合作社、农业企业集中，扩大适度规模经营。加快培育新型经营主体，新发展家庭农场200家、合作示范社100家、市级农业龙头企业20家。大力发展“一村一品、一乡一业”。推动牡丹、木瓜的种植，提高产业化水平。发展山药、芦笋、罗汉参、黄金桃等特色种植。力争标准化基地面积达到420万亩，设施蔬菜种植面积达到110万亩。抓好农产品质量安全县创建，争取认证“三品一标”农产品150个以上。开展粮改饲试点，支持农作物秸秆“过腹还田”，提高粮食就地转化率，新增省级以上畜禽标准化示范养殖场30家。深入实施“一村一品”百村示范、千村推进工程，新增市级以上“一村一品”示范村镇50个以上。四是繁荣发展现代服务业促转型。大力发展商贸物流业。突出抓好各类专业批发市场和济铁物流园、传化物流园等项目建设，壮大商贸服务集群。大力发展电子商务与实体流通相结合的物流体系，实现商业模式的创新和提升。鼓励支持商贸流通企业将物流产业链条向农村延伸覆盖，加快构建市县乡村四级物流体系。力争社会消费品零售总额突破1500亿元，商贸物流业交易额达到2800亿元、增长9%以上。深入挖掘“一都四乡”文化底蕴，大力发展文化产业。繁荣发展文化旅游业，实现旅游消费总额120亿元。加快发展现代金融业。争取银行及非银行类金融机构在我市设立分支机构，新增保险公司3家，规范发展民间融资机构10家。依托生物医药产业优势，着力发展大健康产业。抓好新媒体广告产业园、智能物流园建设，加快形成新的增长极。五是大力发展电子商务促转型。积极实施全市电子商务发展三年行动计划，围绕推动由点向面、由量向质、由线下向线上转变，实施网络提速降费行动，设立“电商基金”，抓好电子商务示范园区建设，扶持壮大电商平台和电商企业，推动互联网与制造业、加工业融合发展，借助互联网促进企业技术创新、经营模式创新，催生“新实体经济”蓬勃发展。实现“千县万村”全覆盖，培训电商人才1万人，电子商务交易额达到1000亿元。六是加快发展开放型经济促转型。坚持引资与引智引技有机结合，带动外贸结构优化。抓好青岛保税港区菏泽功能区建设，启动菏泽综合保税区申报工作，放大公用型保税仓库、直通式海关监管场站运营效应，提高贸易便利化水平。抓好外贸出口转型升级基地、出口农产品质量安全示范区建设。加快培育跨境电商经营主体，提高本地企业属地报关率。力争完成进出口50亿美元，实际利用外资2.5亿美元。

　　（三）完善城市功能，推进基础突破，为经济社会发展提供强有力支撑。以创建省级文明城市为契机，转变城市发展方式，完善城市治理体系，提高城市治理能力，加快建设和谐宜居、富有活力、独具特色的现代化城市。一是竭尽全力推进重大基础设施建设。菏泽机场加快建设，鲁南快速铁路客运通道曲阜经菏泽至兰考段、德上高速巨野经成武至单县段、枣菏高速菏泽段开工建设；东明黄河公路大桥改造完成，东新高速和丹阳立交桥竣工通车；菏徐铁路菏泽段、郓城新河航道菏泽段、国电菏泽电厂四期、华电定陶电厂、大唐郓城电厂、华润菏泽电厂二期等项目取得明显进展；升级改造境内G240牡丹区至河南界等国道140公里，济董路等省道190公里。二是下大气力提升市区规划建设管理水平。围绕增强市区的辐射带动能力，全面提高城市工作的系统性。强化规划引领。按照“多规合一”要求，统筹考虑城市发展，尽快完成城市总体规划的修编报批，抓紧启动城市综合交通、绿地水系、地下管廊、海绵城市等专项规划。着力完善城市框架体系。围绕拓展城市发展空间，年内开工建设长江路东延穿日南高速直通德商高速工程，北外环东延跨赵王河、京九铁路连接上海路工程，广州路北延跨京九、赵王河、日东高速连接菏鄄路工程，广州路南延跨万福河大桥连接定陶县工程，西安路南延跨新石铁路、万福河连接日南高速工程，中华路西延连接水库西二路配套工程，连接菏鄄路至刘民路的国花大道工程等。切实提升城市的通透性和微循环能力。改造提升昆明路从北外环至220国道段、广福街从中华路至东方红大街段、丹阳路从人民路至天香路段、人民路从长江路至定陶界路段、曹州路从和平路至天香路段，打通东方红大街从西安路至重庆路段、八一路从牡丹路至双河路段，建设赵王河天香路桥、康庄路桥。全力推进棚户区改造。用足用活各级支持棚户区改造的政策措施，实施棚户区改造项目111个、6.2万户。加大市区棚改力度，牡丹区完成棚改项目22个、13278户，开发区完成11个、7350户，高新区完成1个、2138户。加快实施符合条件的老旧小区改造。积极推进城市生态文明建设。加快赵王河上游公园、赵王河下游湿地公园综合开发建设，推进老城区环城公园提档升级、延河路雨污水工程，加强洙水河、环城公园、小黑河等水体综合治理。三是统筹推进县城和小城镇建设。鼓励各县区充分利用新型城镇化政策，注重提升内涵，有序推进“三个市民化”。抓好省级示范镇和市级重点镇建设，推动产城融合。推进农村新型社区建设。巩固城乡环卫一体化成果，推进生态文明乡村建设。

　　（四）深化重点领域改革，推进体制机制创新，不断激发内生发展动力。一是行政审批制度改革。继续推进审批服务“两集中、两到位”，精简压缩审批环节和办理时限，深化商事制度改革，进一步优化营商环境。推进政务服务规范化建设，加快建立市县网上政务服务平台。二是财税金融体制改革。严格编制部门预算，实施预算管理改革，推进公开进程。深化国库集中支付、政府购买服务改革，进一步健全“四位一体”公共资源市场化配置管理体系。全面推开“营改增”，支持企业实行主辅分离，将服务业从主业里剥离，形成独立纳税主体，确保应收尽收。推动国税、地税服务深度融合，执法适度整合、信息高度聚合，不断推进税收征管体制和征管能力现代化。加快发展现代金融业，探索建立市级互联网支付中心。完成各级党政机关公务用车制度改革。三是企业改革。推动民营企业建立现代企业制度，促进规模以上企业进行规范化公司制改造，实现由工厂制向规范化公司制转变，完成企业股改50家。深化国有企业改革，支持市属国有企业发展混合所有制经济。四是城市管理体制改革。深入推进城市执法体制改革，完善“两级政府、三级管理、四级网络”城市管理体制，实现城市管理重心下移、力量下沉。鼓励企业和市民通过多种方式参与城市建设、管理，实现城市共治共管、共建共享。创新城市管理考核机制，进一步完善城市管理和服务。五是农村综合改革。抓好农村土地确权登记颁证成果验收和应用，推进农村产权交易服务平台建设，加快农村集体产权制度改革步伐。积极开展农村土地承包经营权抵押贷款、贷款担保业务。以土地托管、领办合作社为切入点深化供销社改革。六是社会事业改革。深化基础教育综合改革，全面推开中小学校长职级制和去行政化。启动城市公立医院综合改革，推动分级诊疗工作开展，逐步放开医疗服务价格。鼓励社会资本兴办养老机构，推进医疗卫生事业、大健康产业和养老服务业融合发展。

　　（五）凝聚各方力量，落实精准措施，坚决打赢脱贫攻坚战。将脱贫攻坚作为头等大事来抓，以更大的决心和超常规的力度，全力加快脱贫进程，确保年内完成40万人脱贫。一是压实脱贫责任。围绕2018年全面完成脱贫目标，坚持“六个精准”和“五个一批”，科学编制脱贫攻坚规划，细化分解年度脱贫任务，层层签订责任书，健全考核体系，强化督导调度，逐级压实脱贫责任。二是进一步提高脱贫的精准性。坚持对症下药、精准滴灌、靶向治疗。认真组织开展精准识别、建档立卡“回头看”，进一步摸清贫困人口底数、致贫原因、脱贫需求。健全各级精准扶贫脱贫信息平台和数据库，实行动态管理，实现扶贫信息联网共享。分类采取就业创业扶贫、电商扶贫、光伏扶贫、基础设施扶贫、社会兜底扶贫等脱贫措施，努力增强脱贫实效。三是大力推进产业扶贫。实施产业推进工程，增强群众内生发展动力。充分发挥新型经营主体作用，使更多贫困户进入产业化经营链条，分享经营增值收益。四是着力构建大扶贫格局。动员各行各业、各部门及社会各界广泛参与，汇聚多元帮扶的强大合力。

　　（六）坚持民意导向，加强民生建设，努力让人民群众有更多的获得感、更强的幸福感。牢固树立共享发展理念，积极回应百姓关切，努力增进人民福祉，切实让群众过上更好生活。一是扎实做好“基础民生”，为广大群众提供基本生活保障。全面完成农村饮水安全工程，加强农贸市场快检设施建设，保障群众“舌尖上的安全”。推进农村公路网化工程，改造农村公路1000公里。大力实施全民参保登记计划。扎实推进“银龄安康”工程，为老年人幸福生活提供保障。二是扎实做好“普惠民生”，为广大群众提供全覆盖的基本公共服务。完善“三级就业创业服务体系”，新增城镇就业6.5万人，转移农村劳动力10万人。加快推进“全面改薄”工程，积极化解城镇普通中小学大班额，推动教育均衡发展。大力引进、膨胀优质教育资源，支持社会力量办学；推进驻地高校改革，支持高等教育发展；整合职教资源，启动职教园区建设，支持职业教育发展；加快特殊教育发展步伐，满足特殊群体的学习需求。巩固完善基本药物制度和基层运行新机制，加快实施基本公共卫生服务项目，开展农村适龄妇女“两癌”免费检查和贫困“两癌”妇女救助工作。实施全面两孩政策，继续优化出生人口性别比。大力推进基层综合性文化服务中心、农村文体广场建设。加快推进市体校新校区建设，深入开展全民健身运动。启动市残疾人康复中心建设。用心做好“留守儿童”、“空巢老人”关爱工作。三是扎实做好“底线民生”，让生活困难的群众享受到政府和社会的关怀和帮助。进一步提高社保救助标准，城乡低保标准分别提高到每月400元和每年3000元，五保人员集中、分散供养标准分别提高到每年4400元和每年3000元。抓好城乡困难群体住房保障工作，开工建设城市公租房1876套、完成农村危房改造5000户以上。四是集中力量办好以下10个方面的民生实事。（1）完成全民健身中心、老干部活动中心、老年大学的主体工程建设。（2）开工建设以博物馆、科技馆、工人文化宫、青少年宫、妇女儿童活动中心、艺术馆、城市展览馆为主体的菏泽市市民中心。（3）在市区规划建设20个便民市场、20个停车场、20个健身广场、10个夜市、10个早市。（4）新增加新能源公交车100辆、公交线路5条，新增加公共自行车1000辆、站点50座。（5）新建农村校舍150万平方米，新建、改扩建城区中小学校72所，新建农村幼儿园200所，公开招录教师2000名以上。在乡镇推进乡村教师周转宿舍建设。（6）在市区中华路市实验小学和二十一中处各建一座人行天桥。（7）将职工医保年最高支付限额由20万元提高到50万元、居民医保年最高支付限额由45万元提高到65万元；医疗救助最高标准提高到2.2万元，重特大医疗救助最高标准提高到2.5万元。（8）新建城市社区老年人日间照料中心60处、农村幸福院400处。（9）加快城乡电商服务网络建设，新建城市社区智能投递终端50个、农村电商服务站1000个。（10）淘汰取缔城区燃煤锅炉、黄标车、燃煤烧烤点，消除城区裸露土地，新增集中供热覆盖面积500万平方米，新增环卫保洁面积500万平方米。

　　（七）坚持底线思维，强化红线约束，切实提高可持续发展水平。一是坚定不移提升生态环境质量。完善环境质量生态补偿机制，落实属地管理和行业管理双重责任，实行环境违法案件挂牌督办、约谈、有奖举报、一票否决等制度，构建多元监管共治体系。改扩建现有污水处理厂，提高处理能力，切实解决污水直排和河道污染问题。以对人民高度负责的精神，把大气污染防治放在更加突出位置，采取更加切实有效的措施，提高城市空气质量改善幅度，增加蓝天白云天数。二是坚定不移推进节能降耗。深化能效对标，加大节能改造，着力淘汰落后设备和工艺，确保实现节能量20万吨标煤以上。三是坚定不移节约高效利用资源。强化水资源消耗、建设用地等总量和强度“双控”。四是坚定不移防范化解金融风险。加强社会征信体系建设，优化金融生态环境。加强风险监测预警，稳妥处置化解不良资产和担保圈风险，打击非法集资活动和恶意逃废银行债务行为，坚决守住不发生系统性和区域性风险的底线。五是坚定不移抓好安全生产。完善安全生产责任体系，扎实抓好重点领域、重点行业的专项整治，坚决杜绝重特大安全事故发生。六是坚定不移维护社会和谐稳定。深化“平安菏泽”、“法治菏泽”建设，着力构建立体化社会治安防控体系。加强矛盾纠纷排查化解。健全公共法律服务体系，提高依法解决群众诉求的能力和水平。

　　五、切实加强政府自身建设

　　各位代表，做好今年的各项工作，任务艰巨，责任重大。我们将牢记宗旨、恪尽职守，自觉践行“三严三实”要求，全面加强自身建设，努力打造人民满意政府。

　　（一）强化学习研究，提升能力素质。建立健全政府机关全员学习制度，定期举办专题培训班和系列讲座，全面加强新理论、新政策、新知识的学习。深入开展政府领导干部集中学、带头学活动，端正学风，提高实效。鼓励各级各部门结合岗位实际，积极开展各类业务培训，拓宽工作思路，推动工作创新，切实增强干事创业的能力和水平。

　　（二）强化依法行政，规范权力行为。自觉运用法治思维和法治方式深化改革，推动发展。坚持“法无授权不可为、法定职责必须为”，时刻做到心中有法、行必依法，严格按照法定权限和程序行使权力、履行职责。严格执行人大及其常委会的决定、决议，自觉接受监督，定期报告政府工作。在重大决策前、决策中主动加强与人民政协的民主协商。扎实办理人大代表建议和政协提案。认真听取民主党派、工商联、无党派人士、人民团体的意见，广泛接受新闻媒体和社会各界监督，确保权力在阳光下运行。

　　（三）强化责任担当，推动工作落实。经济发展进入新常态，精神面貌要有新状态。增强直面问题的担当意识，勇于担负起新一轮发展的重任，把责任和使命扛在肩上。对认准的目标、承担的责任、份内的工作，知难而进，迎难而上，一件一件抓落实，一项一项求突破，一个时间节点一个时间节点向前推进，多为干成想办法、不为干不成找借口，锲而不舍抓到底，不见成效不罢休。健全目标责任体系，严格考核奖惩措施，加强效能监察，严肃问责问效，着力克服庸懒散现象。

　　（四）强化廉洁从政，打造清廉政府。认真落实党风廉政建设责任制，严守政治纪律和政治规矩，任何时候都不“破底线”、不“闯红线”，筑牢拒腐防变的“铜墙铁壁”。巩固拓展党的群众路线教育实践活动成果，健全完善“三严三实”作风建设长效机制。深入基层、深入群众，建立民意导向机制，扎扎实实为群众办实事解难题。完善行政监察，强化财政资金使用、工程招投标、公共资源交易等重点领域、关键岗位的审计监督，坚决纠正损害群众利益的不正之风，努力打造一支政治坚定、能力过硬、勤勉尽责、清正廉洁的人民公仆队伍。

　　各位代表，新的任务艰巨光荣，新的征程催人奋进。让我们在中共菏泽市委的坚强领导下，凝聚全市人民的智慧和力量，以更加饱满的热情、更加坚定的信心、更加务实的作风、更加有效的措施，开拓创新、攻坚克难，齐心协力、扎实苦干，为早日实现“科学赶超、后来居上”的宏伟目标、夺取全面建成小康社会决胜阶段的全面胜利而努力奋斗！
